# The Contribution of Plant Dioxygenases to Hypoxia Signaling

**DOI:** 10.3389/fpls.2020.01008

**Published:** 2020-07-08

**Authors:** Sergio Iacopino, Francesco Licausi

**Affiliations:** ^1^ Department of Biology, University of Pisa, Pisa, Italy; ^2^ Department of Plant Sciences, University of Oxford, Oxford, United Kingdom; ^3^ Institute of Life Sciences, Sant’Anna School of Advanced Studies, Pisa, Italy

**Keywords:** dioxygenase, hypoxia, phytohormone, cell signaling, proteolysis

## Abstract

Dioxygenases catalyze the incorporation of one or two oxygen atoms into target organic substrates. Besides their metabolic role, these enzymes are involved in plant signaling pathways as this reaction is in several instances required for hormone metabolism, to control proteostasis and regulate chromatin accessibility. For these reasons, alteration of dioxygenase expression or activity can affect plant growth, development, and adaptation to abiotic and biotic stresses. Moreover, the requirement of co-substrates and co-factors, such as oxygen, 2-oxoglutarate, and iron (Fe^2+^), invests dioxygenases with a potential role as cellular sensors for these molecules. For example, inhibition of cysteine deoxygenation under hypoxia elicits adaptive responses to cope with oxygen shortage. However, biochemical and molecular evidence regarding the role of other dioxygenases under low oxygen stresses is still limited, and thus further investigation is needed to identify additional sensing roles for oxygen or other co-substrates and co-factors. Here, we summarize the main signaling roles of dioxygenases in plants and discuss how they control plant growth, development and metabolism, with a focus on the adaptive responses to low oxygen conditions.

## Introduction

Dioxygenases comprise a large family of specialized enzymes that catalyze the incorporation of two oxygen atoms into an organic substrate or a primary organic substrate plus a co-substrate. In this redox reaction, four electrons are provided by the substrate or two by the substrate and two by an external donor (Bugg and Ramaswamy, 2008). The activity of dioxygenases results in a variety of modifications including dioxygenation, epoxidation, hydroxylation, and oxidative cleavage of C-C bonds, which affect plant metabolisms, physiology, and development.

Thanks to the vast set of reactions catalyzed by dioxygenases, the control of their activity constitutes a powerful tool for future biotechnological applications in plants. To date, dioxygenases have been effectively exploited in phytoremediation processes (Hernández-Vega et al., 2017) and development of biosensor (Iacopino et al., 2019). Moreover, from a synthetic biology perspective, their dependency on common cellular substrates and cofactors allows the transfer of dioxygenases between unrelated organisms (Iacopino et al., 2019; Masson et al., 2019; Puerta et al., 2019), opening possibilities towards the development of novel regulatory networks conferring new agronomical traits and improving fitness under challenging abiotic conditions.

The requirement of molecular oxygen as a co-substrate inevitably link dioxygenases activity to plant response to chronic and acute hypoxia, which is caused by ambient oxygen limitation, such as in the case of submergence or waterlogging, or as part of developmental programs (Weits et al., 2019). However, the affinity of each dioxygenase enzyme for oxygen and the protein expression levels can compensate for co-substrate shortage. Here, we summarize the different subfamilies of plant dioxygenases and their role of in plant metabolism and development, discussing their involvement and potential application under low oxygen stress.

## Subfamilies of Plant Dioxygenases

Since dioxygen is a stable diradical triplet, it requires to be activated before reacting with organic substrates. To attain oxygen activation, most dioxygenases use a non-heme iron cofactor located into the catalytic site of the enzyme. The type of iron cofactor and its coordination, together with the requirement of additional co-substrates, allows the classification of dioxygenases into six subfamilies (Mitchell and Weng, 2019).

1. Catechol dioxygenases, comprising both non-heme iron-dependent intradiol and extradiol dioxygenases, mediate the conversion of catechol substrates in ring-opened products with the incorporation of oxygen into aldehydes and carbonylated functional groups (Bugg and Ramaswamy, 2008). Intradiol dioxygenases utilize a ferric cofactor coordinated by two His and two Tyr residues (Ohlendorf et al., 1988) while extradiol dioxygenases bind a ferrous cofactor through two His and one Asp/Glu residues (Hegg and Que, 1997). Both types of dioxygenases do not require additional co-substrates as electron sources (Wang et al., 2017).
2. Lipoxygenases (LOX) are a group of non-heme iron dependent dioxygenases that catalyze the conversion of polyunsaturated fatty acids to hydroperoxides. In these proteins, the iron is coordinated by three His residues and the carboxyl terminal group of the protein (Andreou and Feussner, 2009).
3. The Rieske dioxygenases compose the smallest family of plant dioxygenases and catalyze the dihydroxylation of arene substrates (Mitchell and Weng, 2019). Together with oxygen, a NADH molecule is required as cofactor for the reaction. In this subfamily of enzymes, a 2Fe-2S cluster acts as a bridge to transfer electrons from the NADH to the catalytic site, where the iron atom is coordinated by two His and one acidic residue (Gibson et al., 1970).
4. Carotenoid cleavage dioxygenases (CCDs) mediate the cleavage of alkene chain in various tetraterpenoids. Differently from the other non-heme iron-dependent dioxygenases, the Fe atom in these enzymes is coordinated by four His residues whereas no carboxylate residue is involved (Harrison and Bugg, 2014).
5. Thiol dioxygenases catalyze the oxidation of the sulfur atom present in amino acid derivatives to their corresponding sulfinates. Green plants possess two types of thiol dioxygenases: cysteine dioxygenase (CDO), typical of green algae, which catalyze free cysteine catabolism and N-terminal plant cysteine oxidase (PCO), present also in land plants (Weits et al., 2014; Hildebrandt et al., 2015). While no information is available for PCO enzymes yet, in CDO proteins the Fe atom is coordinated by three His residues, a feature unique to this subfamily of dioxygenases (Joseph and Maroney, 2007).
6. Finally, 2-oxoglutarate (2OG)-dependent dioxygenases (2-ODDs) represent the largest family of plant dioxygenases with over 130 genes in Arabidopsis (Kawai et al., 2014) involved in a wide range of catalytic functions. The enzymes belonging to this subfamily utilize the ferrous cofactor, coordinated by the canonical two His and one acidic residue', and one acidic residue and 2-OG molecule to incorporate an atom of oxygen into the substrate. The second oxygen atom is instead transferred to the 2-OG molecule with the consequent production of succinate (Martinez and Hausinger, 2015).

These dioxygenases can participate to hypoxic signaling at two levels: they can generate cellular signals in an oxygen dependent manner, but they can also be the targets of hypoxia sensing, being regulated at the transcriptional or post-transcriptional level, as summarized in **Supplemental Table S1**.

## Dioxygenases Participate to Hormone Metabolism

Hormones are essential signaling molecules that regulate different aspects of plant development and physiology, and thus affect crop yield and response to biotic and abiotic stresses. Plants control hormone balance through the conversion from inactive precursor to active signaling molecules and vice versa. The widespread participation of dioxygenases to hormone homeostasis connects their signaling pathways to the availability of oxygen.

**Figure 1:**
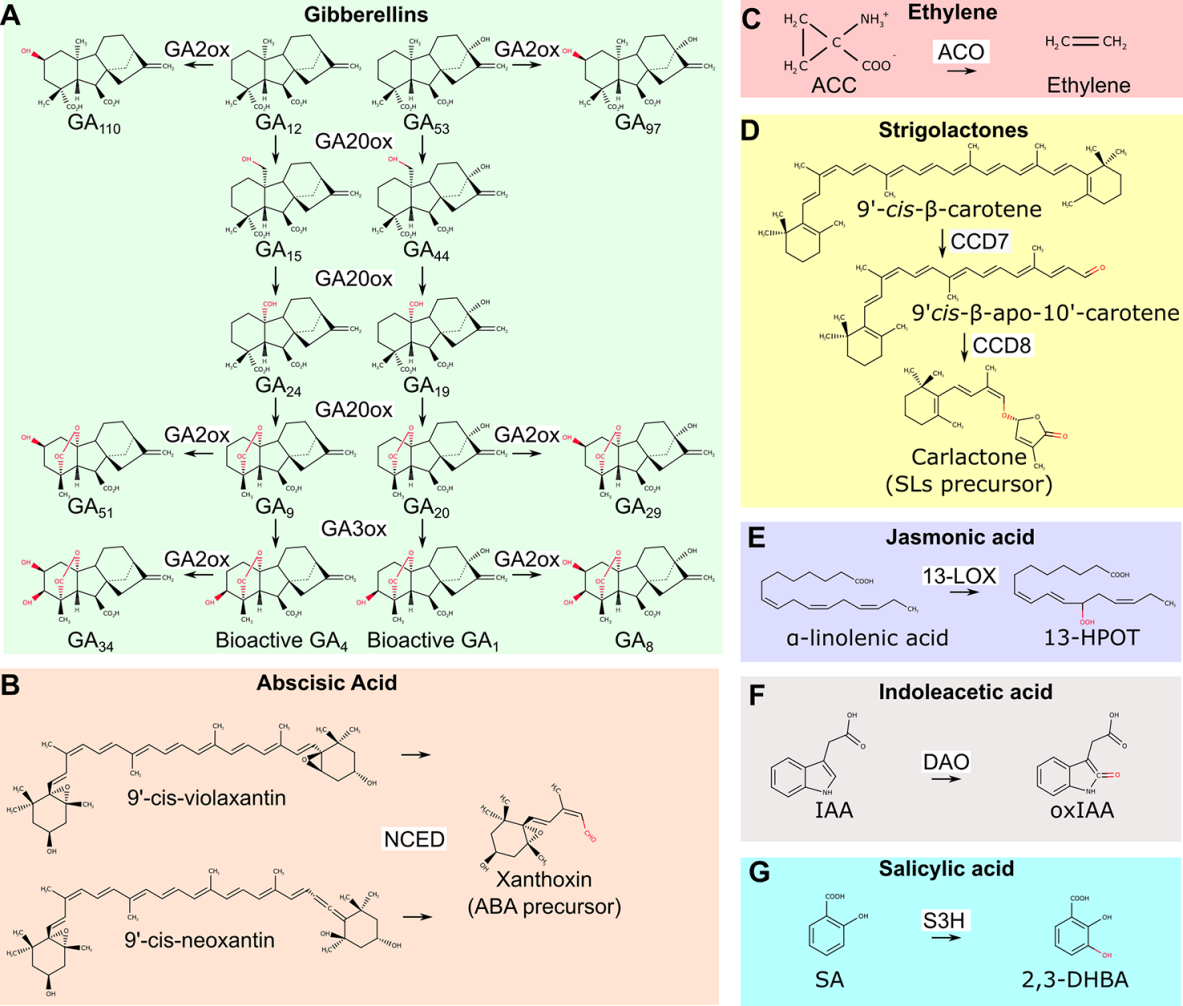
Overview of biosynthetic and catabolic reactions of different hormones operated by dioxygenases. **(A)** GA20ox and GA3ox dioxygenases lead to the production of bioactive GA4 and GA1 molecules. GA2ox mediates their inactivation as well as the inactivation of other biosynthetic precursors. **(B)** The NCED enzyme is required for the production of the biosynthetic ABA precursor xanthoxin. **(C)** The ACO enzyme mediates the final step of ethylene biosynthetic pathway. **(D)** All SLs originates from carlactone, the product of CCD7 and CCD8 dioxygenases. **(E)** Dioxygenation of α-linoleic acid is the first step of jasmonic acid biosynthetic pathway. **(F)** The conversion of bioactive IAA into inactive oxidized IAA is mediated by DAO dioxygenase. **(G)** SA conversion to 2,3-DHBA by S3H dioxygenase. GA20ox, GA20-oxydases; GA3ox, GA 3-oxydases; GA2ox, GA 2-oxydases; NCED, 9-cis-epoxicarotenoid dioxygenases; ABA, abscisic acid; ACC, 1-aminocyclopropane-1-carboxylic acid; ACO, 1-aminocyclopropane-1-carboxylic acid oxidase; CCD7, carotenoid cleavage dioxygenases 7; CCD8, carotenoid cleavage dioxygenase 8; SLs, strigolactones; 13-LOX, 13-lipoxygenase; 13-HPOT, (13S)-hydroperoxyoctadecatrienoic acid; DAO, dioxygenase for auxin oxidation; IAA, indoleacetic acid; IAAox, oxidized indoleacetic acid; S3H, salicylic acid 3-hydroxylase; SA, salicylic acid; 2,3-DHBA, 2,3-dihydrobenzoic acid.

Dioxygenases are involved in the biosynthesis of gibberellins, abscisic acid (ABA), and ethylene (**Figures 1A–C**). The synthesis of gibberellic acids (GAs), phytohormones that regulate plant height, sex determination, and flowering time, requires the action of two 2-ODDs: GA20- and GA3- oxidases, which mediate the conversion of precursors into active GA molecules (Pimenta Lange and Lange, 2006) (**Figure 1A**). A key regulatory step in ABA biosynthesis, the oxidative cleavage of 9-cis-epoxycarotenoids is catalyzed by the CCD 9-cis-epoxicarotenoid dioxygenases (NCED) (Tan et al., 2003) (**Figure 1B**). Finally, the 2-ODD 1-aminocyclopropane-1-carboxylic acid (ACC) oxidase (ACO) catalyzes the final step of ethylene production (**Figure 1C**). Differently from most 2-ODD enzymes, ACO does not use 2-OG as co-substrate, and rather exploits reduced ascorbate as an electron donor (Gómez-Lim et al., 1993).

Interestingly, GAs, ethylene and ABA signaling pathways interact in plant adaptation to flooding. For instance, in wild rice species (*Oryza* spp) these hormones regulate internode elongation and the formation of aerenchymatic tissues. These consist of large air cavities in the root cortex to facilitate gas diffusion. In case of submergence, ethylene concentration raises inside plant tissues to repress ABA biosynthesis and promotes GAs accumulation (Hoffmann-Benning and Kende, 1992; Hattori et al., 2009). Downstream targets of these hormones include genes involved in programmed cell death in the cortex and cell wall remodeling that enable cell expansion (Sasidharan and Voesenek, 2015). In concert, these events stimulate the formation of aerenchyma and promote in parallel rapid shoot elongation (Chen et al., 2010; Parlanti et al., 2011). Therefore, the shoot of submerged plants rapidly elongates, thus escaping from the water level and restoring gas exchange with the atmosphere. The increased synthesis of ethylene and active GAs in different plant species under submergence suggests that the biosynthetic dioxygenases involved in the synthesis of these hormones have a higher affinity for oxygen than its availability in these tissues (Atwell et al., 1988; Hattori et al., 2009). Moreover, low oxygen stresses induces the upregulation of ACC synthase (ACS) and ACO in several plant (Wang and Arteca, 1992; Vriezen et al., 1999; Zhou et al., 2001). Finally, ethylene entrapment due to reduced gas diffusion rate in water, contributes to its local accumulation (Voesenek et al., 1993).

Strigolactones (SLs) are a group of molecules involved in the control of shoot branching, adventitious root development, establishment of symbiotic mycorrhiza and, recently, have been associated to root adaptation to abiotic stress conditions including waterlogging (Bashar, 2018). SLs biosynthetic pathways require the action of two CCD enzymes: CCD7 and CDD8 (Booker et al., 2004; Auldridge et al., 2006). They sequentially regulate the production of the precursor of all SLs, carlactone (Abe et al., 2014) (**Figure 1D**). Genetic inactivation of CCD7 and CCD8 is sufficient to block SLs biosynthesis, which results in a branched shoot phenotype (Gomez-Roldan et al., 2008). Since SLs repress the production of adventitious roots in waterlogged tomato plants, manipulation of CCDs enzymes represents a promising strategy to improve nutrient and oxygen uptake and thus promote plant fitness (Vidoz et al., 2010; Kohlen et al., 2012).

Finally, dioxygeases also participates to the synthesis of jasmonic acid (JA), which is involved in seed germination, pollen development, plant aging and biotic stresses defense. The first step of JA biosynthesis, the dioxygenation of carbon 13 in α-linolenic acid, requires the action of 13-LOX (**Figure 1E**). In Arabidopsis, 13-LOXs genes are upregulated after desubmergence, a condition where JA plays a fundamental role in the adaptation to the restoration of photosynthesis and aerobic metabolisms that cause a burst of reactive oxygen species (ROS) (Yuan et al., 2017; Yeung et al., 2019).

Dioxygenases also play key roles in the catabolism of plant hormones: auxin (IAA), salicylic acid (SA), and GAs catabolism relies on the action of 2-ODDs. IAA is oxidized to the inactive oxindole-3-acetic acid by DAO (**Figure 1F**), S3H, mediates the hydroxylation of SA to the inactive 2,3-dihydrobenzoic acid (2,3-DHBA) and GA2 oxidase is required for GA catabolism (Yamaguchi, 2008; Zhang et al., 2013; Stepanova and Alonso, 2016) (**Figure 1G**).

Overall, the involvement of dioxygenases in hormone homeostasis provides the opportunity for their exploitation as a tool to regulate plant development, anatomy, defense against pathogens, and survival under negative conditions. Specific dioxygenase activity can be manipulated by affecting the expression level of the relative coding gene, genetically altering the catalytic properties of the enzyme, modulating the abundance of cofactors, agonists and antagonists, or a combination of the interventions listed above. For example, pharmacological attempts to modulate the activity of these enzymes has led to the development of chemical inhibitors of GA biosynthesis and catabolism (King et al., 1997; Mander et al., 1998; Rademacher, 2000). In summary, dioxygenases control key aspects of hormone biosynthesis and catabolism, which in turn regulate plant growth and adaptation under challenging conditions. The requirement of oxygen as co-substrate makes dioxygenases a valuable tool for regulating hormone levels under low oxygen conditions such submergence and waterlogging. The identification of enzyme variants with variable oxygen affinity, obtained by mutagenesis or isolated from alternative plant accessions, may support future breeding of crop varieties with optimized sensitivity to the ranges of oxygen concentrations encountered by the plant during submergence.

## Dioxygenases That Operate Post-Translational Modifications

**Figure 2:**
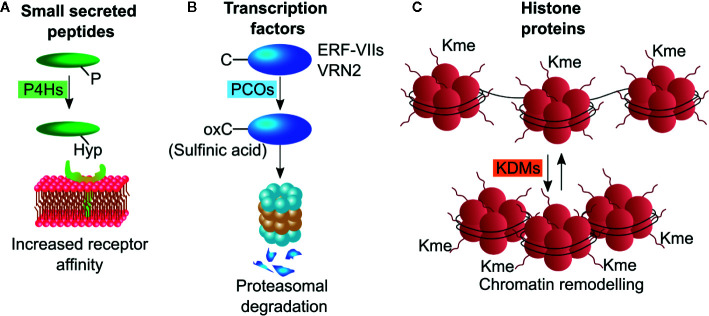
Schematic representations of post-translational modifications operated by plant dioxygenases. **(A)** P4Hs hydroxylate proline residues on small secreted peptides thereby enhancing their receptor affinity. **(B)** PCO enzymes oxidize N-terminally located cysteine residue on ERF-VII and VRN2 transcription factors leading to their degradation through the N-degron pathway. **(C)** KDMs remove methyl groups from lysine residues on the N-terminal tails of histone proteins, thus altering chromatin compactness. P4H, proline hydroxylases; PCO, plant cysteine oxidases; KDM, Jumonji C (JmjC)-domain containing lysine demethylases.

Different classes of dioxygenases operate on protein substrates, to oxidize specific amino acid residues and thus alter the conformation or confer new properties to the polypeptide chain. About two-third of proteinogenic amino acids can be hydroxylated, with proline being the most commonly modified residue. In plants, part of the protein substrates serves a structural purpose, such as cell wall proteins, whose proline hydroxylation is a required for O-glycosylation. Additionally, a large number of proteins involved in signaling events are co or post-translationally subjected to dioxygenase activity. These include, but are not limited to, signaling peptides, enzymes and transcription factors. The specific modification imposed by dioxygenases alters protein features, such as increased or decreased affinity for protein partners, reduced or enhanced stability and ability to bind DNA. Cysteine oxidation, proline hydroxylation and demethylation of histone proteins are the most common posttranslational modifications performed by dioxygenases (**Figure 2**), and thus the targets of most research endeavors.

### Proline Hydroxylation

Prolyl-residues can be hydroxylated at their γ-C atom by 2-ODDs enzymes defined as pro- and asn-4-hydroxylases (P4H) generating hydroxyproline (Hyp). In land plants, proline hydroxylation is frequently found in cell-wall proteins, such as arabinogalactan proteins (AGPs) and extensins (Fragkostefanakis et al., 2012; Fragkostefanakis et al., 2014), and in secreted peptide hormones including CLAVATA3 CLV3, root meristem growth factors (RGFs), and plant peptides containing sulfated tyrosine (PSYs) (Kondo et al., 2006; Amano et al., 2007; Matsuzaki et al., 2010). These small peptides act as short- and long-range signaling molecules regulating stem cell maintenance, proliferation and differentiation (Stuhrwohldt and Schaller, 2019). They originate from longer peptides that are trimmed and subjected to a series of post translational modifications including proline hydroxylation (Matsubayashi, 2011). These modifications are often sequentially linked and appear to be required for full activity of the small peptides (**Figure 2A**). Although some sequence motifs are shared among the small peptides, a clear consensus sequence required for proline hydroxylation could not be identified so far. In mammals, P4Hs control the stability of the hypoxia inducible factor α (HIFα), a transcription factor regulating cellular responses to hypoxia stresses. (Epstein et al., 2001). The Arabidopsis genome contains 13 genes encoding for putative P4Hs. Those that have been tested, show different substrate specificity and subcellular localization, with the majority in the Endoplasmic Reticulum and Golgi Apparatus (Stuhrwohldt and Schaller, 2019). The Arabidopsis At-P4H-1 could effectively hydroxylate a HIFα peptide *in vitro* and some *P4Hs* genes are differentially regulated in Arabidopsis in response to hypoxia (Hieta and Myllyharju, 2002; Vlad et al., 2007), However, endogenous PHDs were unable to hydroxylate a HIF fragment endogenously produced in Arabidopsis (Iacopino et al., 2019).

### Cysteine Oxidation

Cysteine oxidation proceeds through three states where oxygen atoms are progressively bound to the sulfur in the side-chain of the amino acid: sulfenic (-SOH), sulfinic (-SOOH), and sulfonic (-SOOOH), the latter being an irreversible reaction. N-terminal cysteine sulfinylation of specific proteins has been proposed to act as a molecular sensor of NO, O_2_, and heme in animal cells since this post-translational modification labels proteins as substrates for proteasomal degradation by the Arg-branch of the N-degron pathway (Tasaki et al., 2009). Moreover, it was recently shown that animals possess N-terminal cysteine oxidase (NCOs) enzymes able to specifically catalyze the dioxygenation of N-terminally cysteine residues (Masson et al., 2019). Sulfinylated proteins are targets of arginyltransferases (ATE) that conjugate an arginine residue (White et al., 2017). This modification is finally recognized by N-recognins (Ubr1 in animals and PRT6 in plants) that mediate ubiquitination and subsequent targeting of the protein to the proteasome (Garzón et al., 2007) (**Figure 2B**). PCOs and animal aminoethendiol dioxygenase (ADO) share moderate similarity between their amino acid sequences. The number of plant NCO substrates demonstrated experimentally is relatively low: enzymatic *in vitro* cysteine oxidation has been reported exclusively for the group VII of ethylene responsive factors (ERF-VIIs) and the polycomb group protein vernalization 2 (VRN2) (Gibbs et al., 2018) while it remains to be demonstrated for the Arg-degron substrate Little Zipper 2 (ZPR2) (Weits et al., 2019). These transcriptional regulators mediate plant responses to chronic and acute hypoxia, thereby conferring NCOs a role as oxygen sensors in plants and animals (Gibbs et al., 2011; Licausi et al., 2011).

Compatibly with this function, *in vitro* kinetics assays revealed that PCO and ADO have oxygen affinities that allow prompt inactivation under the hypoxic levels know to trigger a response in plants and animals respectively (White et al., 2018; Masson et al., 2019).

Besides hypoxia, conditions that reduce NCO activity such as nitric oxide and low temperature elicit a similar response (Gibbs et al., 2014; Gibbs et al., 2018; Hartman et al., 2019). For instance, stabilization of ERF-VII transcription factors and induction of anaerobic genes in conditions of iron deficiency and zinc excess has been proposed to result from the PCO inactivation that is observed in both conditions (Dalle Carbonare et al., 2019). These observations support the proposed role of plant dioxygenases as metal sensors (Vigani et al., 2013).

### Histone Demethylation

Jumonji C (JmjC)-domain containing lysine demethylases (KDM) constitute a class of 2-ODD enzymes that act on mono-di- and tri-methylated lysine residues in the N-terminal tail of histone proteins and thus play a critical role in defining the accession of the transcriptional complex to promoters (Strahl and Allis, 2000; Lan et al., 2008) (**Figure 2C**). For instance, the increase of the methylation state on K4 on histone H3 (H3K4me3) is associated to gene expression (Yu et al., 2009). Instead, increased methylation state on K9 and K27 of histone H3 is expected to negatively affect gene expression (Yu et al., 2009). The Arabidopsis genome codes for 21 JMJ enzymes, mainly characterized for their involvement in the regulation of flowering time and ABA responses (Gan et al., 2014; Wu et al., 2019).

KDM proteins have been proposed as oxygen sensors in animal cells. Indeed recent assessment of their low affinity for oxygen and consequent inactivation under hypoxia, associated with the widespread increase in histone methylation observed *in vivo* in such condition, confirmed this role (Chakraborty et al., 2002; Shmakova et al., 2014; Hancock et al., 2017; Batie et al., 2019; Chakraborty et al., 2019). A similar role for plant KDM has to be confirmed yet. In rice plants subjected to submergence, the trimethylation state of hypoxic genes was observed to increase significantly (Tsuji et al., 2006). Still, direct evidence of changes in histone methylation associated to a reduced activity of KDMs under hypoxia is missing. Interestingly, four Arabidopsis KDM mRNAs were observed to increase their association with polysomes in hypoxia, suggesting that these are preferentially translated (Branco-Price et al., 2008). Future research efforts will be directed at characterizing the affinity of plant KDMs for oxygen and their susceptibility to chronic and acute hypoxia, as well as the effect of hypoxia on specific histone methylation.

## Future Directions for Research on Plant Dioxygenase

Plant dioxygenases absolve different functions in biological systems, ranging from hormone biosynthesis to epigenetic chromatin rearrangements. Moreover, dioxygenases with low oxygen affinity have been developed by both animals and plants to perceive oxygen levels. The adaptation to environmental conditions that restrict oxygen availability, such as submergence, require changes in hormone profiles, chromatin rearrangements, and modulation of transcription factor activity. All these processes involve the participation of dioxygenases at different levels, thereby directly linking the intensity of the response to the oxygen content. Further structural and biochemical characterization of dioxygenases and the sensitivity of their targets to oxygen limitation will pave the way to deeper understating of molecular processes that enhance survival under such adverse conditions and thus possibly support the generation of crops with increased submergence tolerance.

## Author Contributions

SI and FL wrote the manuscript and conceptualized the figures. SI prepared the figures.

## Conflict of Interest

The authors declare that the research was conducted in the absence of any commercial or financial relationships that could be construed as a potential conflict of interest.
